# Evaluation of a subcutaneous continuous glucose monitoring system in critically ill neonatal foals

**DOI:** 10.1093/jvimsj/aalaf059

**Published:** 2026-01-21

**Authors:** Flavie Payette, Laurence Leduc, Darko Stefanovski, Michelle Abraham, Andrew van Eps

**Affiliations:** Department of Clinical Studies, New Bolton Center, University of Pennsylvania, School of Veterinary Medicine, 382 W Street Road, Kennett Square, PA 19348, United States; Clinical Sciences Department, Faculté de Médecine Vétérinaire, Université de Montréal, Montreal, Quebec, Canada; Department of Clinical Studies, New Bolton Center, University of Pennsylvania, School of Veterinary Medicine, 382 W Street Road, Kennett Square, PA 19348, United States; Department of Clinical Studies, New Bolton Center, University of Pennsylvania, School of Veterinary Medicine, 382 W Street Road, Kennett Square, PA 19348, United States; Department of Clinical Studies, New Bolton Center, University of Pennsylvania, School of Veterinary Medicine, 382 W Street Road, Kennett Square, PA 19348, United States

**Keywords:** continuous glucose monitoring system, dysglycemia, equine, foal, glucometer, hypotension

## Abstract

**Background:**

Dysglycemia is common in critically ill neonatal foals. Continuous glucose monitoring systems (CGMS) are potentially useful in these cases, but factors such as poor peripheral perfusion could interfere with results.

**Hypothesis/Objectives:**

Evaluate the correlation, agreement, and accuracy of CGMS compared to point-of-care glucometry (POCG) and laboratory analysis (LAB) in critically ill neonatal foals and assess the impact of hypotension on CGMS measurements.

**Animals:**

Fifteen critically ill, client-owned neonatal foals.

**Methods:**

In a prospective method comparison study utilizing clinical cases, glucose concentration was measured serially using CGMS and POCG (every 6 h), and LAB (every 24 h) for pairwise comparison. Blood pressure was measured every 12 h.

**Results:**

Average bias (95% limits of agreement) between CGMS and LAB, POCG and LAB, and CGMS and POCG were 48 mg/dL (−27 to 111), 10 mg/dL (−23 to 45), and 38 mg/dL (−21 to 98), respectively. Spearman’s correlation was significant between CGMS and LAB (*r* = 0.65), POCG and LAB (*r* = 0.77), and CGMS and POCG (*r* = 0.75). The CGMS accuracy was low with only 15.5% of CGMS concentrations within 15% of LAB concentrations, compared with 88.3% for POCG. Hypotension did not affect CGMS measurements.

**Conclusions and clinical importance:**

The CGMS provided glucose measurements above both LAB and POCG concentrations. Given its low correlation and accuracy, CGMS cannot replace LAB or POCG as the sole glucose measurement method in critically ill foals. However, it is a useful adjunct for tracking trends and providing alerts.

## Introduction

Dysglycemia is common in critically ill neonatal foals, with only 29% of foals maintaining euglycemia upon hospital admission.^1^ Hypoglycemia (<76 mg/dL) is associated with worse survival, systemic inflammatory response syndrome (SIRS), and positive blood culture results, whereas extreme hyperglycemia (>180 mg/dL) also has been associated with negative outcomes.^1–6^ Sick foals are at increased risk of dysglycemia because of factors such as decreased glucose intake, low glycogen and fat stores, endotoxin-induced insulin resistance or glucose utilization, impaired gluconeogenesis, increased catabolism associated with primary disease, and treatments such as parenteral nutrition, insulin, or glucocorticoids. Evidence from intensive care unit (ICU) studies in humans suggests that decreased glucose variability and more time spent within the euglycemic range are associated with improved outcomes,^7^^,^^8^ and decreased glucose variability is particularly critical in pediatric patients, where time spent outside of the euglycemic range has been linked to neurodevelopmental disorders, increased comorbidities and worse outcomes.^9^

Blood glucose traditionally is measured serially in hospitalized foals, requiring repeated venipuncture and considerable restraint, which can cause stress-induced hyperglycemia. Continuous glucose monitoring systems (CGMS) offer a noninvasive alternative, tracking glucose concentration in interstitial fluid and providing real-time data every 5 min. These systems use a subcutaneous sensor to measure glucose by a glucose-oxidase electrochemical reaction, transmitting an electric signal proportional to the glucose concentration to the receiver via the attached transmitter. Key advantages over point-of-care glucometers (POCGs) include automatic alerts for abnormal glucose concentrations and remote data display, which can help avoid detrimental periods of hypo- or hyperglycemia. Different devices have been evaluated in both adult horses^10–15^ and foals,^10^^,^^16^^,^^17^ showing promising results for monitoring individual glucose trends. Although early studies on CGMS in neonatal foals showed wide limits of agreement (LoA) with standard laboratory assays (LABs) and glucometers, recent data on the Dexcom G6 suggest potential for accurate glucose monitoring in both healthy and ill neonatal foals.^10^^,^^16^^,^^17^ However, concerns remain regarding the accuracy of this type of device in critically ill foals, particularly those with marked hypoglycemia, dehydration, hypotension, or rapid fluctuations in glucose concentrations.

Our objectives were to determine the correlation, agreement and accuracy of the Dexcom G6 CGM in comparison to standard LAB and POCG for monitoring glucose concentrations in critically ill neonatal foals. In addition, the impact of hypotension on interstitial glucose measurements was evaluated. We hypothesized that the CGMS would generate accurate glucose measurements with acceptable agreement with measurements using other methods (POCG and LAB) in critically ill foals, and that the precision of the CGMS would be acceptable for clinical use in hypo- and hyperglycemic and hypotensive foals.

## Materials and methods

### Design and animals

A prospective clinical method comparison study was conducted using a convenience sample of 15 foals. Glucose measurements using CGMS, POCG, and LAB were compared serially over the hospitalization period in all foals. Sample size was considered adequate based on previous studies evaluating similar continuous glucose monitors in foals.^16^^,^^17^ Sick foals 7 days of age or younger and presented to or born at the University of Pennsylvania’s New Bolton Center during the 2022 foaling season were eligible for inclusion with owner consent. Foals presented for failure to thrive, sepsis, neonatal encephalopathy, localized infection, or other conditions were included in the study, unless presented exclusively for flexural deformities or other noninfectious orthopedic conditions. All foals underwent complete physical examination and complete blood analysis, including CBC, serum biochemistry panel (LAB), plasma fibrinogen and serum immunoglobulin G (IgG) concentrations, and POCG upon hospital presentation. Sepsis scores were calculated based on Wong’s updated sepsis score including equine neonatal SIRS criteria (Score 2).^18^ Foals previously diagnosed with a coagulopathy were excluded from the study.

### CGMS placement

The CGMS Dexcom 6 (Dexcom Inc, San Diego, CA) was evaluated. Placement of the CGMS was performed by one of the authors (F.P. or L.L.). Briefly, a 2 × 3-inch area was clipped over the lateral aspect of the hindquarter and cleaned with chlorhexidine scrub followed by isopropyl alcohol.^17^ Once the region was dry, the Dexcom G6 sensor was placed following the manufacturer’s recommendations using the applicator. Rapid drying adhesive glue was applied to the periphery of the application pad and the transmitter was inserted into the sensor. After application of the device, a mandatory 2-h sensor warm-up period was performed based on the manufacturer’s instructions. By pairing the sensor code with the receiver during set-up, blood or capillary glucose concentrations were not required for sensor calibration with the new Dexcom G6 technology (self-calibration). Manual calibration was performed when indicated by the device.

### Glucose measurements

Glucose concentration measured using the CGMS was compared to blood glucose concentration measured using POCG, and LAB on blood samples obtained via direct venipuncture. Initial (T0) glucose measurements were obtained after calibration of the CGMS. Interstitial glucose concentrations obtained using the CGMS were recorded as displayed on the receiver before venipuncture. Blood was obtained by direct venipuncture of the cephalic vein and glucose concentrations were directly measured from the syringe using the POCG (Accu-Chek Guide meter and Accu-Chek Guide Me meter, Roche Diabetes Care, Inc., Indianapolis, IN) or placed in a potassium oxalate/sodium fluoride blood collection tube (BD Vacutainer, Becton, Dickinson and Company, Franklin Lakes, NJ) and refrigerated until analysis using LAB (Vitros XT 3400 Chemistry System, Ortho Clinical Diagnostics, QuidelOrtho Corporation, San Diego, CA) within maximum 6 h of collection.^19^^,^^20^ The LAB measurements were considered the gold standard, using the glucose oxidase-peroxidase method for measurement. The POCG uses the glucose dehydrogenase method for glucose measurement. After T0 measurements, glucose measurements using CGMS and POCG were performed every 6 h, and LAB measurements were performed every 24 h until failure of the CGMS or hospital discharge. The glucose measurement strips were stored according to the manufacturer’s instructions. Noninvasive blood pressures were obtained by indirect oscillometric measurement using a cuff over the coccygeal artery adjusted to the foal’s size every 12 h for the duration of the study using a veterinary multi-parameter monitor (BM3Vet, Bionet America, Inc., Tustin, CA). Blood pressures were measured in triplicate and average systolic, diastolic and mean (mean arterial pressure [MAP]) results were retained for data analysis. Based on a previous study evaluating glycemia in critically ill foals^1^ as well as usual ranges used in our hospital, euglycemia was defined as a blood glucose concentration between 76 and 180 mg/dL (4.2-10.0 mmol/L), as measured by either method (POCG or LAB). Extreme hypoglycemia was defined as glucose concentrations < 50 mg/dL (<2.8 mmol/L). Hypotension was defined as an MAP < 60 mmHg.

### Statistical analysis

Statistical analyses were performed using STATA (StataCorp, College Station TX) and GraphPad Prism (version 10.2.3, San Diego, CA). Normality of the foals’ clinical data and blood pressure measurements was tested using a Shapiro–Wilk test. For descriptive statistics, normally distributed data were presented as mean ± SD, and data that did not follow a normal distribution were presented as median and range. In addition, the number and percentage of measurements within the hypoglycemic, euglycemic, and hyperglycemic ranges were reported for each method.

As an initial exploratory step, correlation among the 3 methodologies was assessed using Spearman’s rank correlation and interpreted according to Chan’s classification for the assessment of medical assays.^21^^,^^22^ Inference statistical analysis of assessing the agreement between the measures was done in 2 steps. First, because of the repeated measures nature of the observations, the differences between the methodologies were assessed using a modified Bland–Altman approach implemented as mixed-effects linear regressions with glucose concentration as the outcome, fixed effect of method of measurement as a categorical variable, and the animals as the random effect.^23^^,^^24^ Time when the sample was obtained and hypoperfusion were included as confounders in the model. Post-hoc agreement was evaluated with pairwise comparison of marginal (model-adjusted) means, and marginal effects. Second and final Lin’s concordance correlation coefficient (CCC) was calculated and interpreted using McBride’s classification.^25^ Final agreement was based on the combined results of the above-described statistical analyses. Given that CGMS measures glucose concentration in a different compartment (interstitium) versus glycemia evaluated using PCOG and LAB, the absolute values are expected to differ, while still retaining a relationship to each other. Mixed-effects linear regression analysis was applied to develop conversion equations to convert CGMS results to LAB and to POCG equivalents, and the conversion formulae were experimentally applied to the CGMS concentrations. Significance was set at *P <* .05.

Following the International Organization for Standardization (ISO) 15197:2013 minimum criteria to address the analytical accuracy of the CGMS and POCG compared to the reference standard LAB, the numbers of CGMS and POCG concentrations within 10% and 15% of the LAB concentrations were calculated.^26^ In addition, the mean absolute relative difference (MARD), quantifying the deviation of glucose concentrations from the reference measurement and expressed as a percentage, was calculated in pairwise fashion, using LAB as the gold standard, or POCG as the standard for the CGMS–POCG pair.^27^

**Figure 1:**
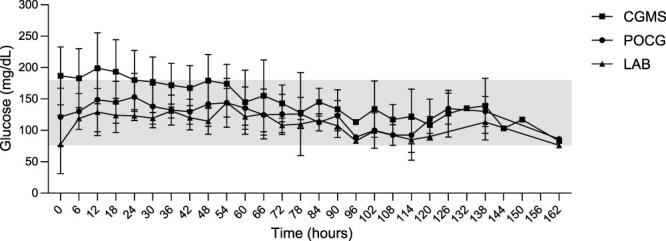
Mean ± SD glucose concentrations over 162 h measured via CGMS (square), POCG (circle), and LAB (triangle) in 15 sick hospitalized foals. The shaded area represents the range of normal glucose concentrations for foals. Abbreviations: CGMS = continuous glucose monitoring system; LAB = gold standard biochemical analyzer; POCG = point-of-care glucometer.

Based on visual graphical analysis showing a larger difference in glucose concentration between the CGMS and POCG or LAB over the first 24-72 h after placement of the device (Figure 1), Bland–Altman analyses comparing bias and 95% LoA from the 3 methods were performed evaluating glucose measurements for the first 24, 48, or 72 h compared to the respective time points (>24 h, > 48 h, or > 72 h) until completion of the study.

## Results

### Foals

Fifteen foals were included in the study. Five were fillies and 10 were colts. Breeds included Thoroughbred (*n* = 4), Standardbred (*n* = 4), Warmblood (*n* = 2), Paint (*n* = 2), Belgian Draft (*n* = 1), Saddlebred (*n* = 1), and Thoroughbred cross (*n* = 1). Median age on presentation was 0.5 days old (range, 0-3 days old). Foals were hospitalized for a median time of 9 days (range, 3-29 days), 13/15 (86.7%) survived to discharge and 2/15 (13.3%) were euthanized in hospital. Median sepsis score was 7 (range, 3-25). Sepsis score was ≥ 12 in 5/15 (33.3%) foals and positive blood cultures were obtained in 3/11 (27.3%) foals, of which two-thirds had sepsis scores > 12. Final diagnoses included (in general more than one diagnosis per foal): failure of transfer of passive immunity (*n* = 12), neonatal encephalopathy (*n* = 6), enteritis/enterocolitis (*n* = 4), omphalitis/urachitis (*n* = 3), prematurity and incomplete ossification (*n* = 2), sepsis (*n* = 2), rib fractures (*n* = 1), pneumonia (*n* = 2), and uroabdomen/bladder necrosis (*n* = 1).

### Sensor performance

The CGMS stayed in place for a mean time of 3.45 ± 1.87 days, and for up to 6.75 days. Three sensors had to be replaced within 48 h of initial installation because of one failure of the device, one dislodgement while the foal was experiencing seizure-like activity and frequent recumbency changes, and one was bitten off by the mare. In one foal with rib fractures, the ribs became acutely displaced and the foal developed hemothorax, at which time the CGMS sensor successfully notified staffing of the foal’s condition because of unreadable concentrations obtained likely secondary to marked hypoperfusion from hypovolemia. For the remainder of the foals, the sensors remained in place and were removed once the foals no longer required intensive monitoring or were ready to be discharged from the hospital. Despite colic episodes, recumbency changes and the foals moving, the sensor stayed in place without issues in these cases.

### Glucose measurements

**Figure 2:**
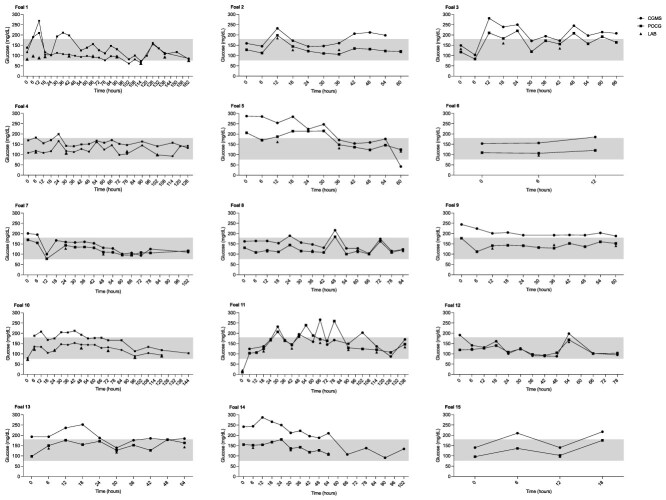
Glucose concentrations over time for individual foals measured via CGMS (square), POCG (circle), and LAB (triangle). The shaded area represents the range of normal glucose concentrations for foals. Abbreviations: CGMS = continuous glucose monitoring system; LAB = gold standard biochemical analyzer; POCG = point-of-care glucometer.

The number of sampling times analyzed was 191 for CGMS, 190 for POCG, and 60 for LAB, with a total of 182 measurements overlapping between CGMS and POCG, 58 between CGMS and LAB, and 60 between POCG and LAB. Glucose measurements over the study period ranged from 42 to 287 mg/dL using the CGMS, from 11 to 269 mg/dL using the POCG, and from < 20 to 185 mg/dL using the LAB. Using linear predictions, glucose measurement marginal means (95% CI) were as follows, respective to each device: CGMS 173 mg/dL (163-183 mg/dL), POCG 135 mg/dL (126-144 mg/dL), and LAB 125 mg/dL (116-134 mg/dL), all significantly different from one another (*P* < .001). For interstitial glucose concentrations assessed using the CGMS, 1.0% of results were within the hypoglycemic (<76 mg/dL) range, 61.8% were in the euglycemic range, and 37.2% were in the hyperglycemic (>180 mg/dL) range. For POCG, 1.6%, 88.4%, and 10.0% of measurements, and for LAB, 5.0%, 93.3%, and 1.7% of measurements were within the hypoglycemic, euglycemic, and hyperglycemic ranges, respectively. Glucose concentrations according to each measurement method are summarized in Table 1 and mean ± SD glucose concentrations plotted per method over time are presented in Figure 1. Glucose data from individual foals are presented in Figure 2.

**Table 1:** Number of glucose concentration recordings, range, marginal means (linear prediction), and percentage of concentrations within the hypoglycemic, euglycemic, and hyperglycemic ranges using 3 methods of measurement: CGMS, POCG, and LAB in 15 sick hospitalized foals.

	**CGMS**		**POCG**		**LAB**	
**Number of recordings**	191		190		60	
**Range (mg/dL)**	42-287		11-269		<20-185	
**Glucose measurement marginal means (95% CI) (mg/dL)**	173^a^ (163-183)		135^a^ (126-144)		125^a^ (116-134)	
** % of concentrations within range**						
** Extreme hypoglycemia (<50 mg/dL)**	1.0%	0.5%	1.6%	0.5%	5.0%	1.7%
** Hypoglycemia (<76 mg/dL)**		0.5%		1.1%		3.3%
** Euglycemia (76 mg/dL ≤ *x* ≤ 180 mg/dL)**	61.8%		88.4%		93.3%	
** Hyperglycemia (>180 mg/dL)**	37.2%		10.0%		1.7%	

^a^All marginal means are significantly different from one another (*P* < .001).

Abbreviations: CGMS = continuous glucose monitoring system; LAB = gold standard biochemical analyzer; POCG = point-of-care glucometer.

### Correlation and agreement

**Figure 3:**
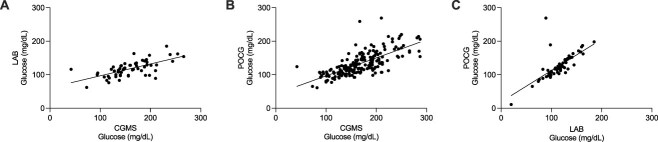
Pairwise display of glucose concentrations (mg/dL) as measured via CGMS and LAB (A), CGMS and POCG (B), and LAB and POCG (C) in 15 sick hospitalized foals. Each dot represents a sampled time point. Spearman’s correlations are presented in Table 2. Abbreviations: CGMS = continuous glucose monitoring system; LAB = gold standard biochemical analyzer; POCG = point-of-care glucometer.

**Figure 4:**
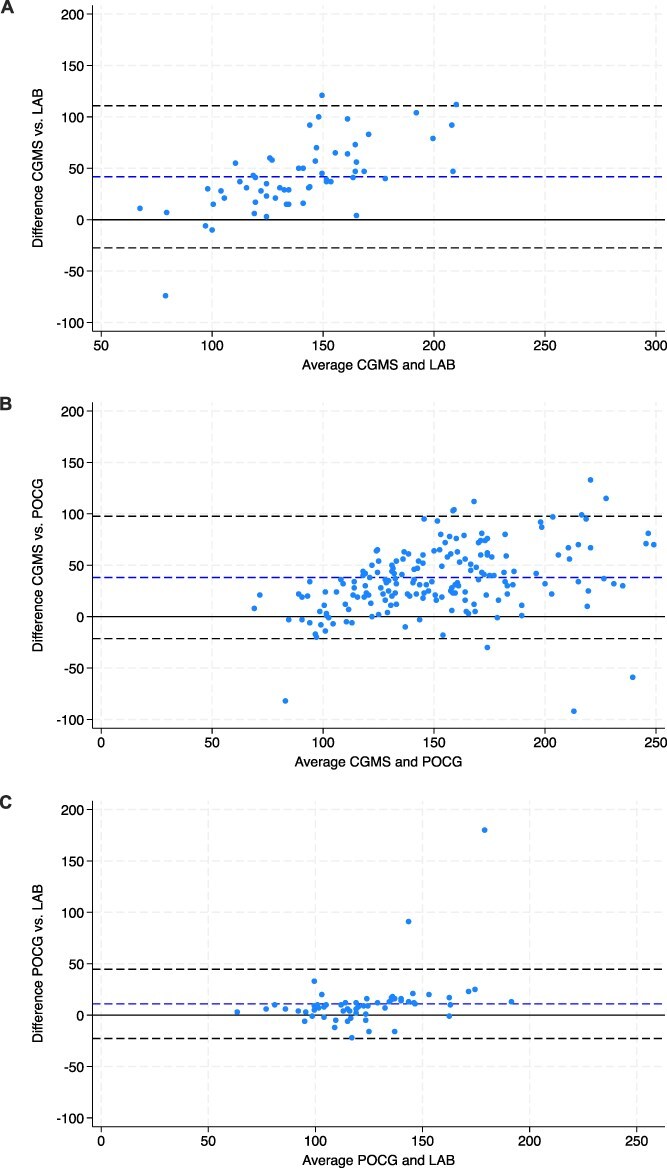
CGMS vs LAB (A), POCG vs CGMS (B), and POCG vs LAB (C) Bland–Altman plots. The central horizontal dashed line represents the average difference between the techniques, and the upper and lower horizontal dashed lines represent the 95% CI. Each dot represents a measurement where both techniques were collected at the same time point. Abbreviations: CGMS = continuous glucose monitoring system; LAB = gold standard biochemical analyzer; POCG = point-of-care glucometer.

Correlations were highest between POCG and LAB (Spearman’s correlation *r* = 0.77, *P* < .001), followed by CGMS vs POCG (*r* = 0.75, *P* < .001), and lowest when comparing CGMS with the gold standard LAB (*r* = 0.65, *P* < .001; Table 2, Figure 3). Mixed-effects models with glucose measurement method as fixed effect, the animals as the random effect, and confounded by time and hypoperfusion were significantly different between CGMS and POCG or LAB (*P* < .001), with no significant effect of time (*P* = .72) or hypoperfusion (*P* = .89). When quantifying the disagreement between methods (concordance), there was a larger interval (contrast) separating the measurements recorded using the CGMS when compared with the 2 other methods. The CGMS consistently provided readings approximately 48 mg/dL (95% LoA, −27 to 111 mg/dL) higher than gold standard LAB concentrations, and 38 mg/dL (95% LoA, −21 to 98 mg/dL) higher than POCG concentrations (Table 2). In contrast, POCG and LAB provided more consistently similar readings, yielding higher concordance (contrast, 10 mg/dL; 95% LoA, −23 to 45 mg/dL). Bland–Altman plots comparing methods in pairwise fashion are presented in Figure 4. Lin’s CCC were poor for all pairwise comparisons, especially when comparing CGMS to LAB (*ρ* = 0.33, *P* < .001; Table 2).^25^ These results show that not only concordance was below the ideal range when comparing 2 methods measuring similar in vivo concentrations but also that the CGMS consistently overestimated glucose concentrations. The POCG had stronger agreement with LAB results.

**Table 2:** Pairwise comparison of glucose concentrations (mg/dL) for the assessment of correlation, agreement and accuracy between 3 methods: CGMS, LAB, and POCG in 15 sick hospitalized foals.

	**CGMS vs LAB**	**CGMS vs POCG**	**POCG vs LAB**
**Number of overlapping results**	58	182	60
**Correlation**			
** Spearman correlation coefficient (*r*) (*P* value)**	0.65 (*P* < .001)	0.75 (*P* < .001)	0.77 (*P* < .001)
**Agreement**			
** Contrast (mg/dL)**	48	38	10
** 95% limits of agreement (mg/dL)**	−27 to 111	−21 to 98	−23 to 45
** Lin’s concordance correlation coefficient (*ρ*) (*P* value)**	0.33 (*P* < .001)	0.50 (*P* < .001)	0.54 (*P* < .001)
**Accuracy**			
** Concentrations within 10% of LAB**	6/58 (10.3%)	NA	35/60 (58.3%)
** Concentrations within 15% of LAB**	9/58 (15.5%)	NA	53/60 (88.3%)
** Mean ± SD ARD (%)**	38.60 ± 26.35	30.64 ± 21.65	14.18 ± 27.73

Positive contrasts reflect an overestimation of the glucose concentration between methods, whereas negative contrasts reflect an underestimation of the measurement.

Abbreviations: ARD = absolute relative difference; CGMS = continuous glucose monitoring system; LAB = gold standard biochemical analyzer; NA = non-applicable; POCG = point-of-care glucometer.

### Corrected CGMS concentrations

The following conversion formula (Table S1) was used to convert CGMS to LAB concentrations: GlucoseLAB = 0.3 × GlucoseCGMS + 70.7. The following formula was used to convert CGMS to POCG concentrations: GlucosePOCG = 0.5 × GlucoseCGMS + 40.3. Bland–Altman plots of corrected concentrations are provided in Figure S1. When compared to clinical results obtained, 97% (58/60) of LAB-corrected CGMS concentrations and 90% (162/182) of POCG-corrected CGMS concentrations fell within the same glycemic range categorization (hypoglycemia vs euglycemia vs hyperglycemia). For comparison, before CGMS correction, only 68% (41/60) and 68% (124/182) of CGMS concentrations were within the same glycemic range of LAB and POCG, respectively.

### Accuracy

Six of 58 (10.3%) CGMS measurements and 35/60 (58.3%) POCG measurements were within 10% of the glycemic concentrations measured by the gold standard chemistry analyzer (LAB; Table 2). Nine of 58 (15.5%) CGMS measurements and 53/60 (88.3%) POCG measurements were within 15% of the LAB measurements. For reference, 29/182 (15.9%) and 47/182 (25.8%) of CGMS measurements were within 10% and 15% of POCG measurements, respectively. Analysis of the MARD and median absolute relative difference showed that the POCG was more accurate than the CGMS, but both had large SDs and ranges (Table 2). Based on these results, the CGMS was considered accurate neither analytically nor clinically when compared with the gold standard chemistry analyzer (LAB), whereas the POCG was more accurate when compared to LAB.

### Effect of hypotension on measurements

Sixty-eight MAP measurements were recorded throughout the study period, with a mean ± SD of 73 ± 16 mmHg. Seven of 14 foals with recorded MAP measurements were hypotensive for at least one time point, for a total of 15 time points where hypotension was recorded across all foals for this study period. Hypotension was not a significant confounder (*P* = .89) of interstitial glucose measurement.

### Effect of time on method agreement

Visual inspection of Figure 1 indicated apparently larger differences in glucose concentrations between the CGMS and the 2 other methods during the initial hospitalization, and therefore Bland–Altman analysis of bias was performed at multiple time points. Bias was larger between the CGMS and LAB or POCG during the initial 24, 48, and 72 h of recordings when compared to bias at their respective later time points (>24 h, > 48 h, and > 72 h; Table S2), but with large overlapping LoA. In addition, time was not a significant confounder (*P* = .72) in the mixed-effects model.

## Discussion

### Continuous glucose monitoring and measurement of glucose in foals

The Dexcom G6, although theoretically useful for near-continuous interstitial glucose measurement, did not show sufficient correlation, agreement, or accuracy compared with the gold standard laboratory chemistry analyzer (LAB) and POCG to be recommended as the sole glucose measurement method in critically ill foals. Specifically, CGMS overestimated glucose concentrations by approximately 48 mg/dL (LoA, −27 to 111 mg/dL) compared with LAB and 38 mg/dL (LoA, −21 to 98 mg/dL) compared with POCG. A previous study reported a bias of 1.6 mg/dL (LoA, −29.4 to 32.6 mg/dL) between the Dexcom G6 and chemistry analyzer in 4 ill neonatal foals.^17^ Similarly, another study obtained a bias of −1.8 mg/dL (LoA, −70.2 to 63.1 mg/dL) between the Guardian REAL-Time CGMS and chemistry in 7 sick foals.^16^ Comparable results with acceptable bias and large LoA were obtained with other CGMS models in healthy and sick adult horses.^10^^,^^12^^,^^13^ In our study, Lin’s CCC between CGMS and LAB was poor (*ρ* = 0.33) and correlation was moderate (Spearman’s *r* = 0.65), whereas a previous study found a higher Lin’s CCC (*ρ* = 0.67) and stronger correlation (*r* = 0.83) in sick foals.^17^ According to the International Organization for Standardization, at least 95% of CGMS results should be within 15% of LAB concentrations (for blood glucose concentrations > 100 mg/dL) to be considered analytically accurate.^26^ In our study, only 15.5% of CGMS concentrations met this criterion, compared with 85% in a previous study.^17^ The MARD was 38%, also indicating poor accuracy. The consistent bias, large LoA, moderate correlation, and limited accuracy compared to LAB measurements in our population preclude recommendation of the Dexcom G6 in critically ill foals as the sole method of glucose monitoring.

In our study, glucometry (POCG) outperformed the CGMS. When compared to the gold standard laboratory chemistry (LAB), POCG showed moderate correlation (*r* = 0.77), stronger agreement (bias, 10 mg/dL; LoA, −23 to 45 mg/dL), poor Lin’s CCC (*ρ* = 0.54) and higher accuracy (88% of concentrations within 15% of LAB). Although accuracy did not meet ideal criteria used in humans (ie, 95% of concentrations within 15% of LAB), and CCC and correlation were moderate to poor, the bias with LAB was clinically acceptable and more tolerable than with the CGMS. These findings contrast with a previous study, where glucometry was less accurate than the CGMS, showing poor accuracy with only 49.5% of concentrations within 15% of LAB, and poor agreement when compared to chemistry, showing results on average 22 mg/dL above, along with wide LoA (−9.3 to 53.67 mg/dL).^17^ Previous studies of equine neonates and adults using similar glucometers (Accu-Chek or Accutrend Plus) found less than ideal agreement with laboratory chemistry, particularly when using whole blood samples, which showed clinically relevant differences and poor correlations.^28–30^ The glucometer tended to underestimate measurements by 20 mg/dL versus chemistry readings in neonatal foals,^29^ and in adult horses, whole blood results showed a bias of up to 45 mg/dL when compared with chemistry results, usually along with wide LoA. Sample type used for glucometry affected results, with anticoagulated (EDTA)^30^ and plasma^28^ samples showing higher correlations and improved bias and LoA compared with standard chemistry in equine samples. Many factors have been noted to influence glucometry results in ICU settings in human medicine, such as changes in hematocrit, metabolic acidosis, hypoperfusion and altered oxygen tensions, but these factors were not evaluated in our study. A veterinary-specific glucometer (AlphaTRAK) showed high accuracy when using equine whole blood samples,^31^ but the same device was found to be less accurate in neonatal foals in a previous study.^17^ In our study, whole blood was used with a glucometer designed for humans to reflect usual emergency protocol in our hospital, which could explain the differences in POCG and chemistry results. Nonetheless, POCG outperformed CGMS and had more clinically acceptable differences from LAB.

### Individual foal data

The CGMS allowed for tracking of individual glucose trends and provided alarms when concentrations fell outside of the target range, while decreasing the need for frequent blood collection, patient restraint, and minimizing the associated workload. However, the limited agreement obtained between CGMS and LAB raises the question of whether CGMS interstitial measurements lag behind blood glucose determinations. When comparing CGMS and POCG measurements (Figure 1), both followed similar trends and overestimated glucose concentrations compared with LAB, which produced lower results. The LAB glucose measurements were not analyzed instantly compared with POCG samples; samples were stored in potassium oxalate/sodium fluoride tubes and refrigerated until analysis. Although this type of blood collection tube often is viewed as the gold standard for glucose measurement, glycolysis still can be noted in equine samples if analysis is not performed rapidly, and on average a 13 mg/dL decrease in concentration can be noted over 24 h when stored at room temperature.^32^ Rapid changes in glycemia are reported for human blood samples if not refrigerated or centrifuged promptly.^19^^,^^20^^,^^33^^,^^34^ In our study, samples were refrigerated immediately but not centrifuged until analysis, usually within 3 h of collection. This delay may have slightly decreased LAB glucose concentrations, but it is unlikely to account for the substantial bias between LAB and CGMS or POCG measurements. Assuming our gold standard accurately reflected glucose concentrations, both POCG and CGMS overestimated glucose concentrations, with CGMS showing a higher bias. Although visual assessment of individual foal data (Figure 2) did not indicate a notable lag between CGMS and glycemia, POCG was measured every 6 h and LAB measurements were taken once daily. Rapid glucose fluctuations between these intervals were not closely monitored or confirmed with paired LAB analyses. Interstitial glucose concentrations do not change instantaneously, leading to a lag between CGMS and blood glucose concentrations, particularly during rapid changes. In healthy horses with induced hypoglycemia, CGMS lagged 10 min behind POCG, whereas during induced hyperglycemia, the lag increased to 20 min.^13^ In humans, using the Dexcom G6, the mean lag between blood and interstitial glucose concentrations was 4.5 min.^35^ Other CGMS studies in horses showed correlations with glycemia and fairly accurate blood glucose fluctuations.^10–12^^,^^14^^,^^15^ Considering that the CGMS measures interstitial glucose concentrations whereas LAB and POCG measure blood glucose concentrations, it is expected that glucose concentrations will differ between the 2 compartments. To correct for this factor, correction formulae were applied to the CGMS data, with promising results obtained. Overall, CGMS allowed for tracking of glucose trends and demonstrated sensitivity to glucose changes, and although it likely required time to adjust to changes, lag time did not seem to be the reason for positive bias between CGMS and LAB in our study. The application of a corrective formula to CGMS concentrations improved the classification of glucose measurements within the same glucose range of both LAB and POCG, and this approach could improve the agreement and accuracy of the CGMS for clinical use.

### Improvement of agreement over time

Visual analysis of individual trends and bias calculation showed that the CGMS glucose measurements overestimated LAB measurements more markedly during the first 48-72 h after CGMS placement compared with later time points, although with overlapping LoAs. A similar trend was observed previously within the first 30 h of placement using the same device, with improved correlation to gold standard measurements at later stages.^17^ In humans, this phenomenon is poorly understood but often attributed to internal calibration, influenced by variations in sensor sensitivity because of interactions with the interstitial environment and immune system reaction.^35–37^ In our study, equilibrium was reached at later time points, suggesting additional factors might be influencing CGMS measurements. Foreign body reactions (FBRs) to the sensor in host tissues (including local inflammation, protein and platelet adsorption, macrophage infiltration, and fibrous capsule formation) can impair sensor performance as early as 7 days after placement by decreasing its ability to measure interstitial glucose concentrations.^37–41^ Although no studies have investigated this specific issue in horses, the decrease in bias seen in our study 48-72 h after implantation may be a consequence of early FBR resulting in sensor performance degradation, making initial overestimations falsely align more closely with LAB. Similarly, microdialysis probes inserted in the dermis and lamellae of horses led to local inflammation and subsequent tissue healing within 120 h.^38^ Although long-term CGMS implantation is less concerning in sick neonatal foals compared with human patients, the stronger bias observed during the initial 48-72 h of implantation in our study remains poorly understood and complicates product validation within the time frame of its potential usefulness in this population.

### Continuous glucose monitoring in critical patients (hypotension, hypoglycemia, and shock)

Decreased glucose variability and increased time spent in the euglycemic range have been associated with improved outcomes in human ICU patients.^7^^,^^8^ Current glucose monitoring guidelines primarily rely on intermittent measurements obtained from arterial blood samples using the laboratory chemistry or blood gas analyzer.^8^ Point-of-care glucometers, particularly those using capillary blood samples, are considered insufficiently accurate and may introduce errors in glycemic measurements in critically ill patients because of rapid fluctuations in hematocrit, oxygenation and other patient-related factors.^8^^,^^42^ The CGMS offers several potential advantages in critically ill ICU patients, including the ability to monitor trends in blood glucose concentrations, detect glycemic excursions, and intervene before glucose concentrations reach unacceptable ranges. However, concerns persist regarding the validity and accuracy of this type of device in critically ill patients, particularly those with impaired tissue perfusion and rapid changes in glucose concentrations. One study unexpectedly found that CGMS had higher accuracy, but below recommended standards, in patients with septic shock or sepsis compared to those without sepsis.^27^ In patients with distributive shock, CGMS detected glycemic excursions outside the normal range 12.4 times more reliably than intermittent glucose monitoring.^43^ In addition, the use of vasopressors, the presence of edema, and hypotension did not significantly affect CGMS measurements.^44^^,^^45^ Despite the theoretical advantages of CGMS for improving glycemic control, current devices remain insufficiently accurate to be adopted in the ICU setting for human patients as the sole method of glucose monitoring.^7^^,^^27^^,^^43–47^ These devices may be suitable for guiding alarm systems, but are not yet reliable enough to guide therapeutic decisions.

Despite our objective of including critically ill foals with documented episodes of hypotension and hypoglycemia, only 15 of 190 measurements were recorded during concomitant hypotension, and < 5% of recordings fell within the hypoglycemic range (blood glucose concentration < 76 mg/dL). Hypotension did not influence interstitial glucose concentrations when compared to blood glucose concentrations, thereby refuting the hypothesis that a lag would be observed associated with decreased peripheral and interstitial perfusion where the subcutaneous CGMS cannula is placed, altered glucose metabolism resulting from hypoperfusion and inflammation, and peripheral edema. In a previous study, the agreement between CGMS and laboratory chemistry also was found to be higher in ill neonatal foals than in healthy foals.^17^ The high performance of CGMS in patients with presumed decreased peripheral perfusion could be attributed to increased capillary permeability, facilitating the diffusion of intravascular glucose into the interstitial space.^27^ Overall, consistent with the limited number of studies evaluating the effect of hypotension or shock on CGMS accuracy, episodes of hypotension, as measured using noninvasive methods, did not significantly affect glucose measurements in our small number of critically ill foals.

### Limitations

Major limitations of our study include the small number of hypotensive and hypoglycemic events recorded, the infrequent collection of LAB samples (only every 24 h), the different compartments in which glucose concentrations were measured and compared, and the delay in running gold standard LAB tests on these samples. The minimal number of hypoglycemic events may be attributed to foals being stabilized and receiving a dextrose infusion by the time the CGMS warm-up period was being completed. In addition, the CGMS algorithm tends to favor the detection of hyperglycemia, and its accuracy remains clinically insufficient for hypoglycemic ranges, particularly in nondiabetic human patients.^48^ A larger sample size with more frequent CGMS, LAB, and POCG recordings would have been useful in this heterogeneous population. Other factors, such as dehydration and the impact of primary disease processes could have been evaluated further. Furthermore, the Dexcom G6 self-calibration was performed during the initial few hours of hospitalization when the foals theoretically were the most unstable hemodynamically. The self-calibration function was developed for humans and based on our results likely would need modification for foals. Stronger agreement and correlation between the devices may have been observed if the CGMS had been calibrated with POCG at least once daily.

### Conclusion

The Dexcom G6 CGMS did not show sufficient agreement and accuracy when compared to the gold standard chemistry analyzer and POCG to be used as the sole glucose measurement method in critically ill foals. The CGMS did identify individual patient glucose trends without the need for frequent blood collection, animal restraint or increased staff workload and showed improved accuracy in the classification of glycemia when a corrective formula was applied, but insufficient agreement with POCG and LAB limits its utility for guiding therapeutic decisions.

## Supplementary Material

aalaf059_Supplemental_Figure_Tables

## Off-label antimicrobial declaration

The authors declare no off-label use of antimicrobials.

## Institutional animal care and use committee or other approval declaration

All animal manipulations were performed in accordance with the guidelines of the Office of Animal Welfare, and the protocol was approved by the Animal Care and Use Committee of the University of Pennsylvania (POAP # 807248).

## Abbreviations

CCC: concordance correlation coefficient
CGMS: continuous glucose monitoring system
FBR: foreign body reaction
ICU: intensive care unit
ISO: International Organization for Standardization
LAB: gold-standard biochemical analyzer
LoA: limits of agreement
MAGE: mean amplitude of glycemic excursions
MAP: mean arterial pressure
MARD: mean absolute relative difference
POCG: point-of-care glucometer
