# Supplementary material for: Evaluation of a subcutaneous continuous glucose monitoring system in critically ill neonatal foals
Source: J Vet Intern Med. 2026 Jan 21;40(1):aalaf059. doi: 10.1093/jvimsj/aalaf059 (PMC12881958; doi:10.1093/jvimsj/aalaf059)
Supplement: aalaf059_Supplemental_Figure_Tables [file aalaf059_supplemental_figure_tables.zip › Figure S1.docx]

**Figure S1.** Continuous glucose monitoring system (CGMS) vs. gold-standard biochemical analyzer (LAB) (A) and CGMS vs. point-of-care glucometer (POCG) (B) Bland-Altman plots for linear regression-corrected CGMS concentrations. The central horizontal dashed line represents the average difference between the techniques, and the upper and lower horizontal dashed lines represent the 95% CI. Each dot represents a measurement where both techniques were collected/corrected for the same time point.
