# Supplementary material for: Evaluation of a subcutaneous continuous glucose monitoring system in critically ill neonatal foals
Source: J Vet Intern Med. 2026 Jan 21;40(1):aalaf059. doi: 10.1093/jvimsj/aalaf059 (PMC12881958; doi:10.1093/jvimsj/aalaf059)
Supplement: aalaf059_Supplemental_Figure_Tables [file aalaf059_supplemental_figure_tables.zip › Table_S1_copy_aalaf059.docx]

**Table S1.** Linear regression analysis for the correction of continuous glucose monitoring system (CGMS) concentrations according to gold standard biochemical analyzer (LAB) and point-of-care glucometer (POCG) concentrations.

|  | Coefficient | 95% CI |
| --- | --- | --- |
| **CGMS vs. LAB** | | |
| Alpha | 0.31 | 0.21 – 0.41 |
| Beta | 70.69 | 55.13 – 86.24 |
| **CGMS vs. POCG** | | |
| Alpha | 0.55 | 0.45 – 0.65 |
| Beta | 40.33 | 21.36 – 59.30 |
