# Supplementary material for: Evaluation of a subcutaneous continuous glucose monitoring system in critically ill neonatal foals
Source: J Vet Intern Med. 2026 Jan 21;40(1):aalaf059. doi: 10.1093/jvimsj/aalaf059 (PMC12881958; doi:10.1093/jvimsj/aalaf059)
Supplement: aalaf059_Supplemental_Figure_Tables [file aalaf059_supplemental_figure_tables.zip › Table_S2_copy_aalaf059.docx]

**Table S2.** Bias and 95% limits of agreement for glucose measurements (mg/dL) analyzing the first 24, 48 or 72 hours compared to their respective following time points (> 24h, > 48h, or > 72h) until completion of the study, comparing three measurement methods: continuous glucose monitoring system (CGMS), gold standard biochemical analyzer (LAB), and point-of-care glucometer (POCG).

|  | **CGMS vs LAB** | **CGMS vs POCG** | **POCG vs LAB** |
| --- | --- | --- | --- |
|  | Bias (95% limits of agreement) (mg/dL) | | |
| **Initial 24h** | 58  (1 to 115) | 47  (-17 to 110) | 20  (-59 to 99) |
| **Over 24h** | 34  (-31 to 98) | 29  (-32 to 90) | 6  (-13 to 25) |
| **Initial 48h** | 52  (-5 to 108) | 43  (-18 to 103) | 15  (-51 to 81) |
| **Over 48h** | 30  (-40 to 99) | 23  (-41 to 86) | 6  (-10 to 22) |
| **Initial 72h** | 48  (-22 to 117) | 38.97  (-24 to 102) | 13  (-45 to 71) |
| **Over 72h** | 25  (5 to 55) | 17.93  (-43 to 79) | 6  (-10 to 21) |
